# Long-Term Prognostic Role of Computed Tomography Coronary Angiography for Stable Angina

**DOI:** 10.1007/s11936-020-00818-w

**Published:** 2020-07-15

**Authors:** Mohammed N. Meah, Michelle C. Williams

**Affiliations:** 1 BHF Centre for Cardiovascular Science, University of Edinburgh, Chancellor’s Building, SU305, 49 Little France Crescent, Edinburgh, EH16SUF UK; 2 Edinburgh Imaging facility QMRI, University of Edinburgh, Edinburgh, UK

**Keywords:** Computed tomography coronary angiography, Coronary artery disease, Stable angina, Cardiac imaging

## Abstract

**Purpose of review:**

Chest pain is a common presentation, and there are a wide variety of ways in which it can be investigated and treated. There is growing interest in whether the way we reach a diagnosis of angina can affect the long-term prognosis. In addition to its unparalleled negative predictive value, computed tomography coronary angiography (CCTA) gives anatomical information on the extent and severity of coronary artery disease. This article discusses recent research into the ability of CCTA to predict and improve long-term prognosis for patients with stable angina.

**Recent findings:**

Results from retrospective studies, randomised controlled trials and meta-analyses all suggest that initial investigation with computed tomography coronary angiography confers a prognostic benefit. In addition, the most recent studies have shown that the assessment of plaque burden and plaque constituents is predictive of long-term outcomes.

**Summary:**

Management of stable chest pain should be guided by a CCTA-based approach. Future research should focus on whether incorporating plaque analysis strategies into clinical practice confers additional benefit.

## Introduction

To improve upon prognosis, one must make an accurate diagnosis. The term prognosis was coined by Hippocrates, to mean “foreseeing and foretelling” [1]. When doctors give a prognostic statement, they predict the future course of an individual’s condition. However, in order to precisely predict the prognosis in any individual, it is vital that an accurate diagnosis is made. A diagnosis traditionally identifies a person as having or not having a disease. Accurate diagnosis and acknowledgement that there is a prognostic spectrum dependent on the severity of disease are important and can focus treatments on those who stand to gain the most benefit [2]. There is growing interest in how improved diagnosis can positively impact upon prognosis. In those with chest pain for example, accurate diagnosis of the presence and severity of coronary artery disease has the potential to alter management and improve outcomes. This article will discuss the role of computed tomography coronary angiography (CCTA) in assessing and improving the long-term prognosis of patients with stable angina.

## Improving prognosis for patients with stable angina

To understand how CCTA can affect the long-term prognosis of patients with stable angina, we need to understand how it is managed. Chest pain is a common presenting complaint, contributing nearly 4% of all new consultations to general practitioners [3]. A health survey for England estimated that 14% of men and 8% of women between the ages of 65 and 74 have or have had angina [4]. Whilst large-scale epidemiological studies have shown beyond any doubt that managing risk factors such as hypertension are prognostic, [5, 6]. views on more aggressive treatments are more divided.

Historically, stenoses deemed ‘significant’ (visually or due to the presence of ischaemia on testing) would be treated with revascularisation—a treatment with proven prognostic benefit in acute myocardial infarction [7]. Early studies on coronary artery bypass grafting versus medical therapy support this strategy, though they were conducted in an era before statin therapy [8]. Data from the Reduction in Atherothombosis for Continued Health (REACH) registry also suggested that those with stable angina who had undergone revascularisation appeared to do better than those who did not [9]. However, this was not substantiated in prospective randomised trials. The Optimal Medical Therapy with or without PCI for Stable Coronary Disease (COURAGE) [10]. trial, and more recently, the Initial Invasive or Conservative Strategy for Stable Coronary Disease (ISCHAEMIA) [11]. both found that even in the presence of severe ischaemia, there was no prognostic benefit to utilising revascularisation. Ensuring that those with stable angina are on optimal medical therapy is therefore of critical importance.

Today, there is a plethora of investigations available for patients with stable angina, and each technique has unique benefits. Thus, it can be difficult to decide which test is best as a first line, with even international guidelines presenting conflicting recommendations (Table 1) [4, 12–14]. When selecting a diagnostic test, equal emphasis should be placed on the ability to accurately determine presence or absence of disease, the ability to quantify the severity and extent of disease and the overall impact on patient care and prognosis. CCTA is an anatomical test which can assess the presence and extent of coronary artery disease, identify coronary artery stenoses and characterise the constituents of atherosclerotic plaque. In addition, CTCA can provide additional functional information through the assessment CT fractional flow reserve (CT-FFR). Thus, CCTA offers the opportunity to identify a range of potentially prognostic aspects of coronary atherosclerotic plaque.

**Table 1:** Comparison of guideline recommendations for non-invasive investigation of stable chest pain

	NICE (2016) [4]	ESC (2019) [12]	ACC/AHA (2012) [13, 14]
Initial assessment	Assessment based on clinical likelihood. Heavy reliance on typicality of pain (typical/atypical/noncardiac pain).	Assessment based on pre-test probability and clinical likelihood	Assessment based on pre-test probability and clinical likelihood.
First line	Offer 64-slice CT coronary angiography if pain typical/atypical, or non-cardiac with abnormal ECG.	If likelihood of CAD low/intermediate, suggests CT coronary angiography. If likelihood intermediate/high, suggests non-invasive functional imaging.	If likelihood of CAD low/intermediate, suggest exercise ECG. If exercise ECG not possible or of uncertain significance recommends non-invasive functional imaging.
Second line	If CT results are of uncertain significance or non-diagnostic offer non-invasive functional imaging.	If first-line investigation results are of uncertain significance consider alternative depending on what is available locally.	Recommends CT coronary angiography as an alternative to functional imaging if pre-test probability low/intermediate or stress imaging not possible.
Previous history of CAD	In cases with known CAD and worsening symptoms offer non-invasive functional testing if there is uncertainty about symptom aetiology.	In cases with known CAD and worsening symptoms offer exercise ECG or non-invasive functional imaging.	In cases with known CAD and worsening symptoms offer exercise ECG or non-invasive functional imaging.

*NICE* National Institute for health and Care Excellence, *ESC* European Society of Cardiology, *ACC/AHA* American College of Cardiology/American Heart Association, *CAD* coronary artery disease

Non-invasive functional imaging refers to stress echocardiography, myocardial perfusion scanning and stress perfusion cardiac magnetic resonance imaging

## CCTA to assess the severity and characteristics of coronary artery disease

Multiple studies have established the excellent diagnostic accuracy of CCTA compared to invasive coronary angiography to identify coronary artery stenoses [15, 16]. The highly negative predictive value means that it is particularly useful for ruling out coronary artery disease in patients with low to intermediate pre-test probability [17]. CCTA is also very accurate at excluding high-risk distribution of coronary artery disease (left main stem and/or three vessel disease), with a sensitivity of 95% and specificity of 83% [18]. In the Coronary CT Angiography Evaluation for Clinical Outcomes: An International Multicentre (CONFIRM) registry of 27,125 patients, the presence and severity of coronary artery disease on CCTA were predictive of all-cause mortality [19]. Indeed, the prognostic ability of CCTA has been shown to extend beyond 10 years [20•] However, CCTA can identify both the presence of coronary artery stenoses and the presence of non-obstructive coronary artery disease.

**Fig. 1:**
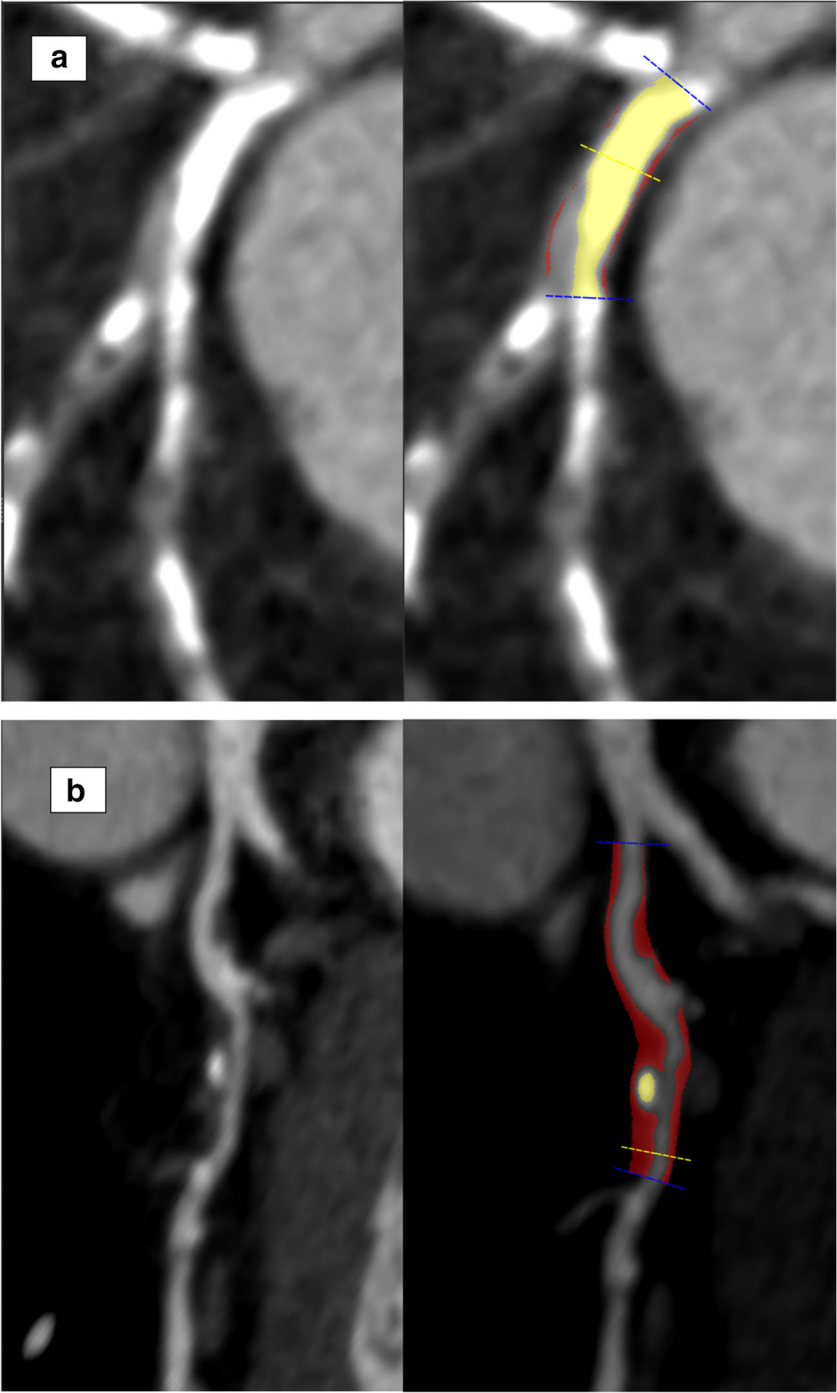
Plaque analysis using CCTA showing **a** an example of calcified plaque and **b** an example of mixed plaque. Calcified plaque is highlighted in yellow and non-calcified plaque is highlighted in red.

CCTA allows accurate quantification of plaque burden which has been shown to improve the ability to risk stratify patients when added to conventional angiographic assessment (Fig. 1) [21]. In a study of 3242 patients undergoing CCTA, Bittencourt et al. showed that the extent of plaque on CCTA enhances risk assessment for patients with both obstructive and non-obstructive disease [22]. For patients with non-obstructive disease in the CONFIRM registry, the extent of coronary artery disease provided more prognostic information than traditional cardiovascular risk factors [23]. CCTA also provides additive prognostic information in older patients (> 70 years old), in whom CCTA has previously been avoided due to the prevalence of heavily calcified plaques. Indeed, the importance of quantifying the extent of nonobstructive disease should not be overlooked, given that a diagnosis of multivessel nonobstructive disease confers a similar prognosis to single-vessel obstructive disease [24].

CCTA not only provides information on anatomy, but can also be used to gain functional information on the significance of coronary artery stenoses. CT-FFR calculated from CCTA using computational fluid dynamic techniques has been shown to improve the accuracy of identifying haemodynamically significant coronary artery disease [25]. In the Prospective LongitudinAl Trial of FFRct: Outcome and Resource Impacts (PLATFORM) study and the Assessing Diagnostic Value of Non-invasive FFR-CT in Coronary Care (ADVANCE) registry, [26]. CT-FFR led to lower rates of invasive angiography showing no obstructive coronary artery disease [27]. Similar to FFR measured during invasive coronary angiography, a CT-FFR above 0.80 has been shown to be associated with a good prognosis [28].

In addition to the burden of atherosclerotic plaque, particularly subtypes of plaque have been shown to confer a worse prognosis. Non-calcified plaque on CCTA is associated with a worse prognosis compared to calcified plaque [29, 30]. Visual assessment can identify high-risk plaque characteristics such as positive remodelling, low attenuation plaque, spotty calcification and the napkin ring sign, which suggest histological instability and are associated with a worse prognosis [31–34]. In the Prospective Multicentre Imaging Study for Evaluation of Chest Pain (PROMISE) trial, the presence of high-risk plaques was associated with an increased risk of major adverse cardiovascular events (hazard ratio (HR) 1.72; 95% CI 1.13 to 2.62, after adjustment for cardiovascular risk and presence of significant stenoses) [35]. However, in the Scottish COmputed Tomography of the HEART (SCOT-HEART) trial, although high-risk plaques were associated with a worse prognosis, this was not independent of coronary artery calcium score, a marker of overall plaque burden [36].

It is now also possible to perform quantitative assessment of atherosclerotic plaque subtypes on CCTA. In the Incidental Coronary EveNts Identified by Computed Tomography (ICONIC) registry, quantitative plaque burden, fibrofatty volume and necrotic core volume on CCTA were associated with risk of subsequent ACS [37]. In the SCOT-HEART trial, quantitatively assessed low attenuation plaque burden was the strongest predictor for fatal and non-fatal myocardial infarction (HR 1.6, 95% CI 1.10–2.34), over and above the presence of coronary artery stenoses or coronary artery calcium score [38•]. These findings challenge our preconceived ideas about the pre-eminence of coronary stenoses and highlight the importance of non-obstructive coronary artery disease in defining prognosis.

## CCTA to clarify the diagnosis

Accurately identifying the presence of coronary artery disease has the potential to alter diagnoses, and subsequently management, for patients with stable angina. Registry studies have established the ability of CCTA to reclassify patients compared to clinical risk scores in up to two thirds of patients [20•]. In the SCOT-HEART [39]. trial, a large multicentre randomised trial assessing the use of CCTA in patients presenting to the chest pain clinic, CCTA clarified the diagnosis in one in four patients [40]. This was further corroborated by Foy and colleagues in their meta-analysis which found that in comparison to functional testing, CCTA led to an increase in coronary artery disease diagnosis and initiation of preventative medications [41]. A further meta-analysis has demonstrated the greater ability of CCTA to exclude atherosclerosis compared to stress testing [41]. By contrast, a negative functional test does not mean the patient is free from non-obstructive coronary artery disease. CCTA therefore not only clarifies the diagnosis, but also identifies those with sub-clinical disease who may benefit from preventive therapies.

## CCTA to improve prognosis

The 5-year outcomes of the SCOT-HEART trial provided the first evidence that management based on CCTA findings could improve clinical outcomes [42••]. In the SCOT-HEART trial, 4146 patients with stable chest pain were randomised to either undergo CCTA or standard care (Table 2). At 5 years, there was a significant reduction in the occurrence of fatal or non-fatal myocardial infarction in patients whose management was guided by CCTA (HR 0.59; 95% confidence interval 0.41 to 0.84; *p* = 0.004). The improved prognosis was not driven by increased revascularisation, but rather, the impact of increased medical therapy, particularly in the group with non-obstructive coronary artery disease [46]. These findings have been replicated in a real-world setting from a national Danish registry [47]. Amongst 32,961 patients who underwent CCTA, there was increased use of preventative medical therapy and a lower risk of myocardial infarction (HR 0.71; 95% CI 0.61 to 0.82) [47]. Interestingly, in the PROMISE study which compared CCTA with functional testing, although there was no difference in outcomes between the two modalities after 25 months, patients with more severe disease had a significantly worse prognosis [45, 48].

**Table 2:** Outcomes from randomised controlled trials comparing an initial strategy of CCTA in patients with stable angina

Trial	*N*	Follow-up	Comparison	Outcome
Randomised Pilot Trial [43] Min et al. (2012)	180	3 months	Myocardial perfusion imaging vs CCTA	• Equivalent improvements in quality of life • Increase incidence of aspirin (22% vs 8%, *p* = 0.04) and statin (7% vs − 3.5%, *p* = 0.03) prescription with CCTA • Lower total cost ($781.08 vs $1214.58, *p* < 0.001) and radiation (7.4 mSv vs 13.3 mSv, *p* < 0.001) with CCTA
CAPP [44] Donnelly et al. (2015)	500	1 year	Exercise ECG vs CCTA	• Improved control of angina symptoms • Fewer patients required further investigations (72 vs 128, *p* ≤ 0.0001) with CCTA • Increased revascularisation and preventative medication with CCTA • Reduced hospital re-attendance (0.8% vs 5.2%, *p* = 0.009)
PROMISE [45] Douglas et al. (2015)	10,003	2 year	Functional test vs CCTA initially	• No difference in primary outcome of major adverse cardiovascular event (3.3% vs 3.0%, *p* = 0.75) at 2 years • Increase in invasive angiography – Less likely to be normal (3.4% vs 4.3%, *p* = 0.02) – More likely to lead to revascularisation (3.2% vs 6.2%, *p* < 0.0001)
SCOT-HEART [39] Newby et al. (2018)	4146	5 year	Standard care vs CCTA	• Significant difference in primary outcome of cardiac death or non-fatal myocardial infarction (2.3% vs 3.9%, *p* = 0.004). • Equivalent rates of invasive angiography and revascularisation by 5 years. • Increased incidence of preventive and anti-anginal therapies.

*CCTA* cardiac computed tomography angiography, *mSv* millisievert

An early criticism of CCTA was the potential to increase the use of invasive coronary angiography. In the PROMISE trial, rates of referral for invasive coronary angiography were higher in patients undergoing CCTA compared to functional imaging, but those undergoing CCTA were less likely to have normal coronary arteries [45]. Furthermore, the 5-year results of SCOTHEART demonstrated that with time, the rate of referral for invasive coronary angiography equalised [42••]. Thus, CCTA can be used to guide more appropriate and timely use of invasive coronary angiography.

Whilst the ability of CCTA to reduce cardiovascular death and non-fatal myocardial infarction has been discussed, one of the CCTA’s greatest strengths is the ability to identify patients with normal coronary arteries. An interesting finding from the SCOT-HEART study was that the greatest quality of life improvements was seen in the cohort of patients who had normal coronary arteries and did not require lifelong medical therapy [46].

Guidelines in the United Kingdom from the National Institute for health and Care Excellence (NICE) are currently the only ones that recommend CCTA as first-line assessment, doing away with pre-test probability calculations (Table 1) [4, 12, 14].. A recent study by Houssany-Pissot and colleagues lends weight to this approach. They assessed nearly 5000 patients who underwent invasive coronary angiography and found that CCTA was a better than functional testing regardless of pre-test probability, limiting unnecessary downstream testing without missing abnormal invasive angiograms [49]. European guidelines recommend CCTA for patients with low/intermediate pre-test probability and we await the updated ACC/AHA guidelines.

## Conclusion

Randomised trials and real-world data show CCTA that can be used to assess long-term prognosis in patients with stable angina. It provides the ability to target optimal medical therapy and prognostic preventative medications to those who will benefit from them. Moreover, invasive management can be directed to those with disease in high-risk locations. In addition, novel techniques can be used to qualitatively and quantitatively characterise high-risk plaques. Management based on CCTA has been shown to improve long-term outcomes and this is reflected in national and international guidelines.
